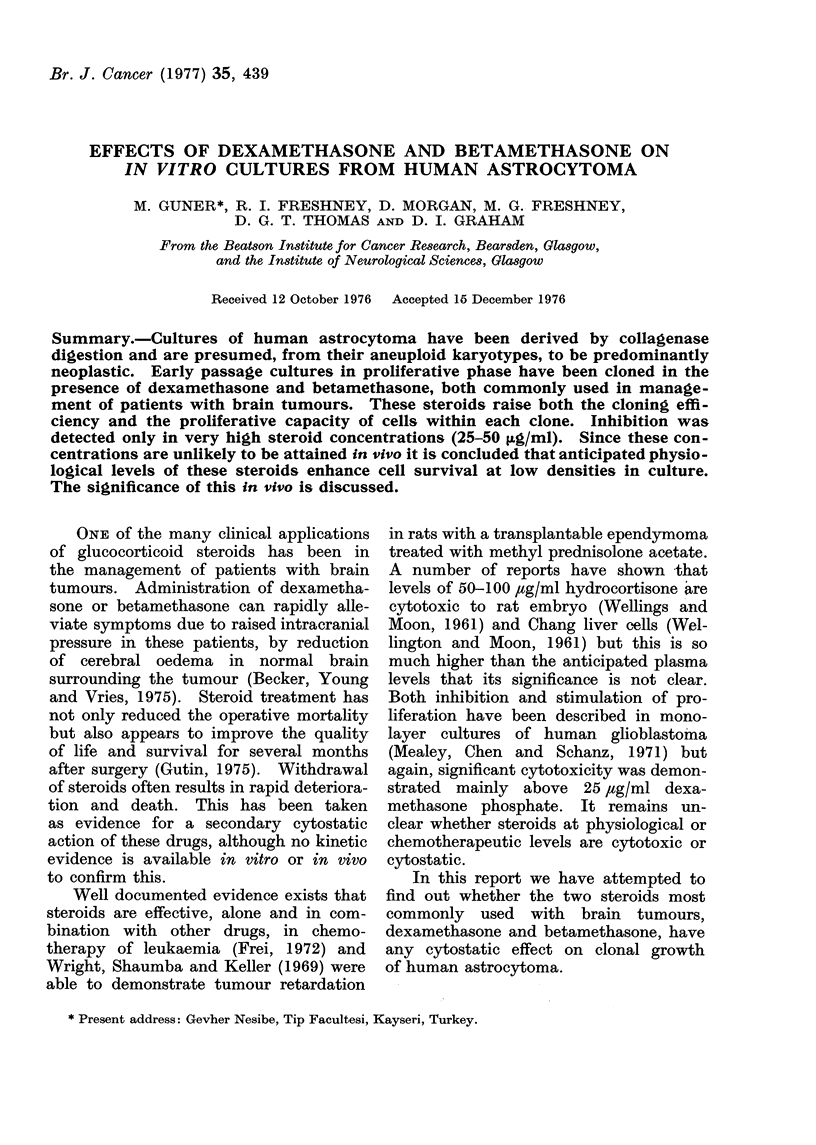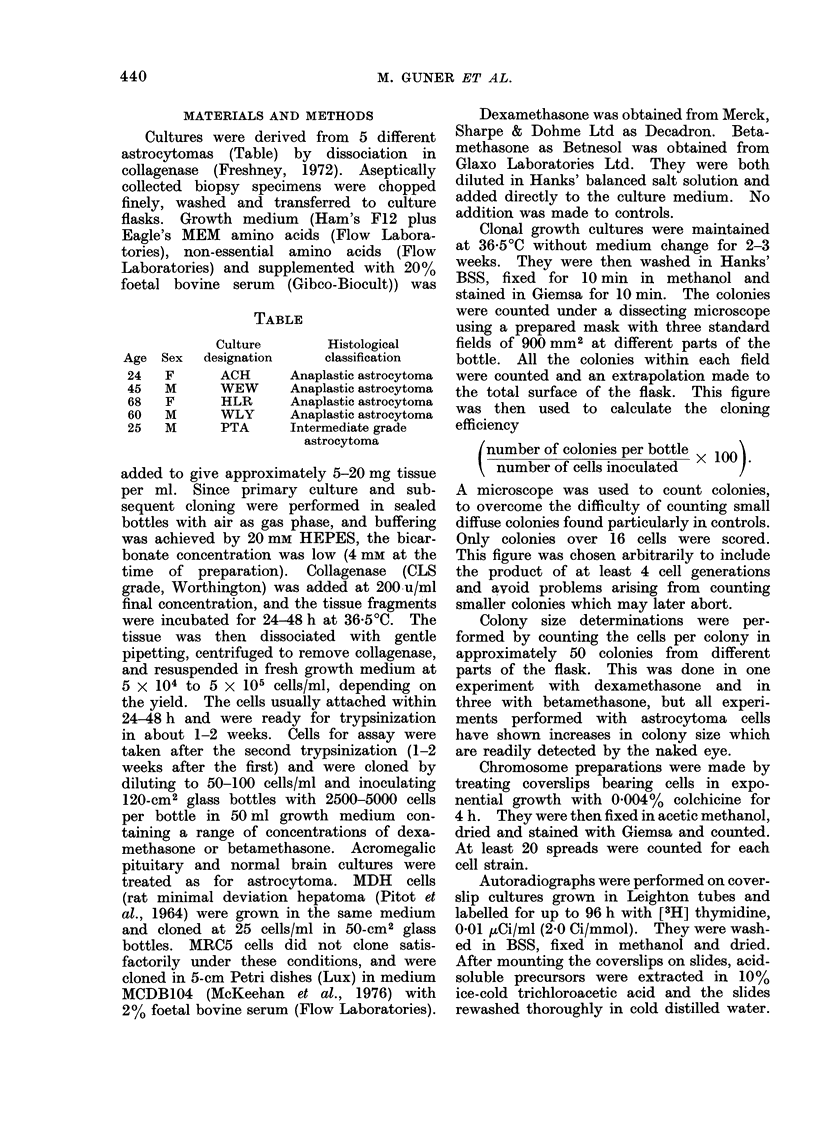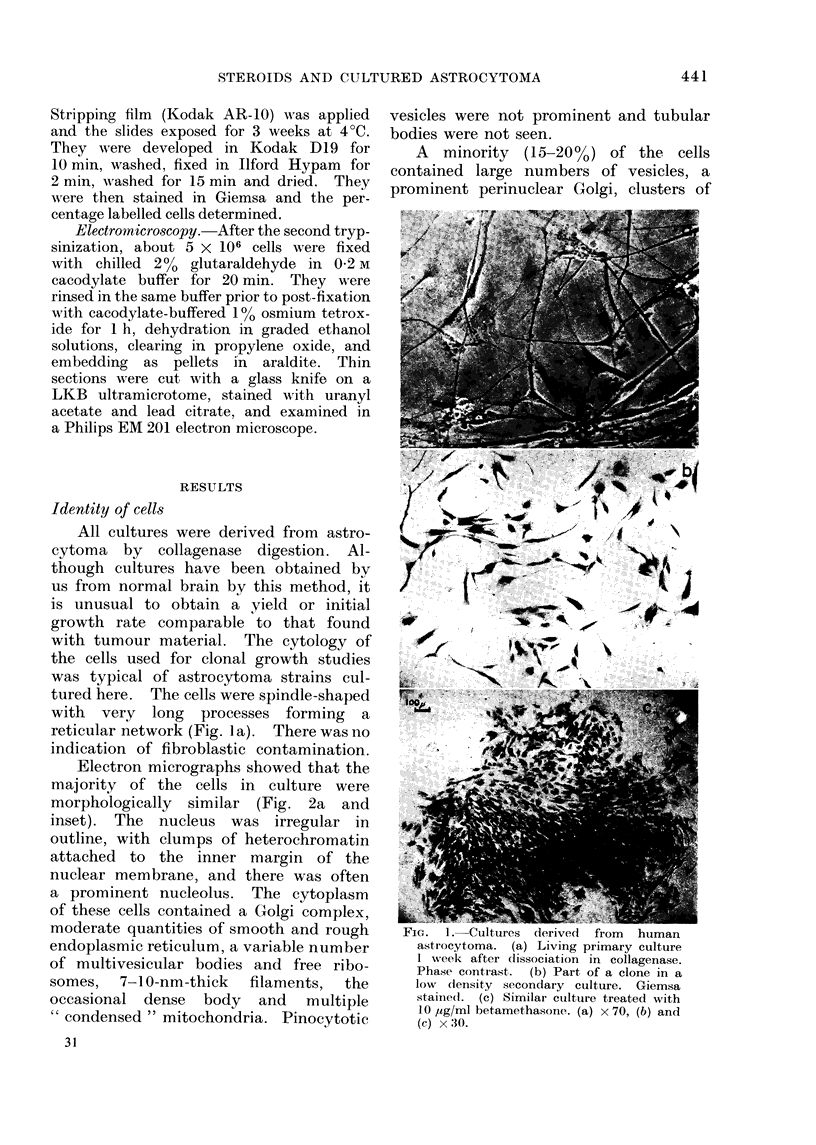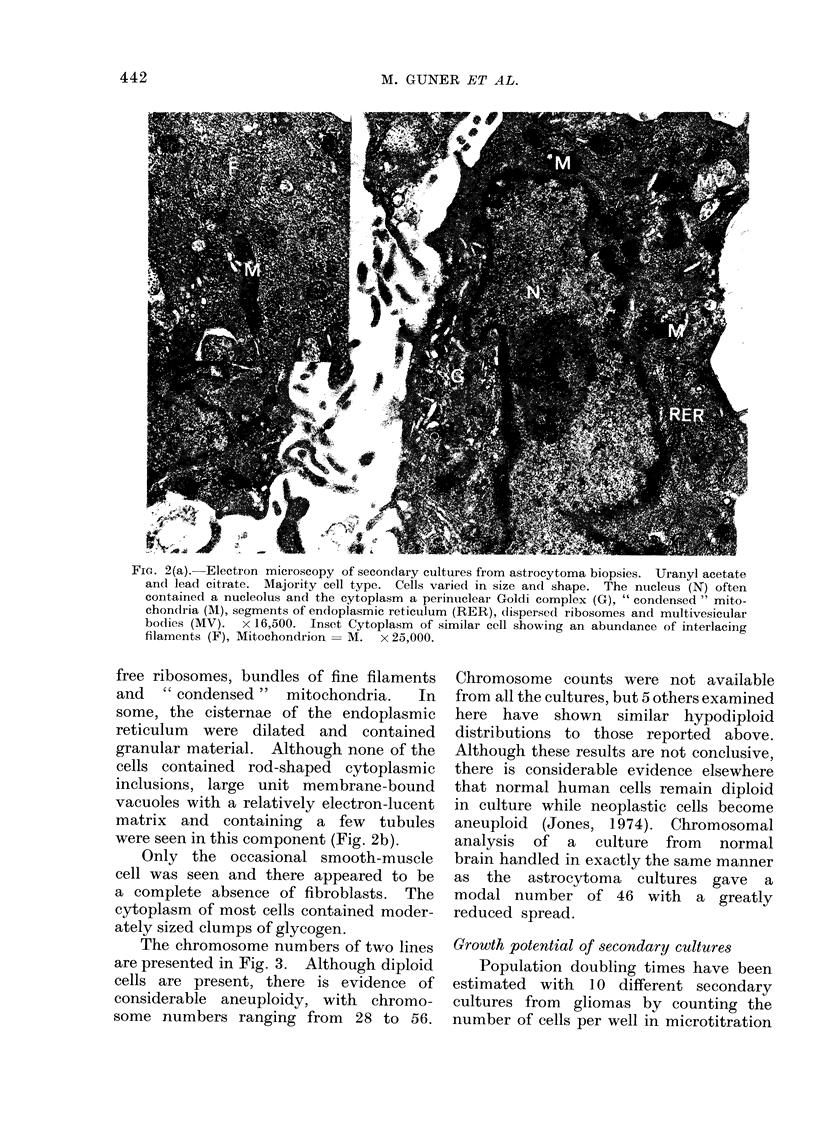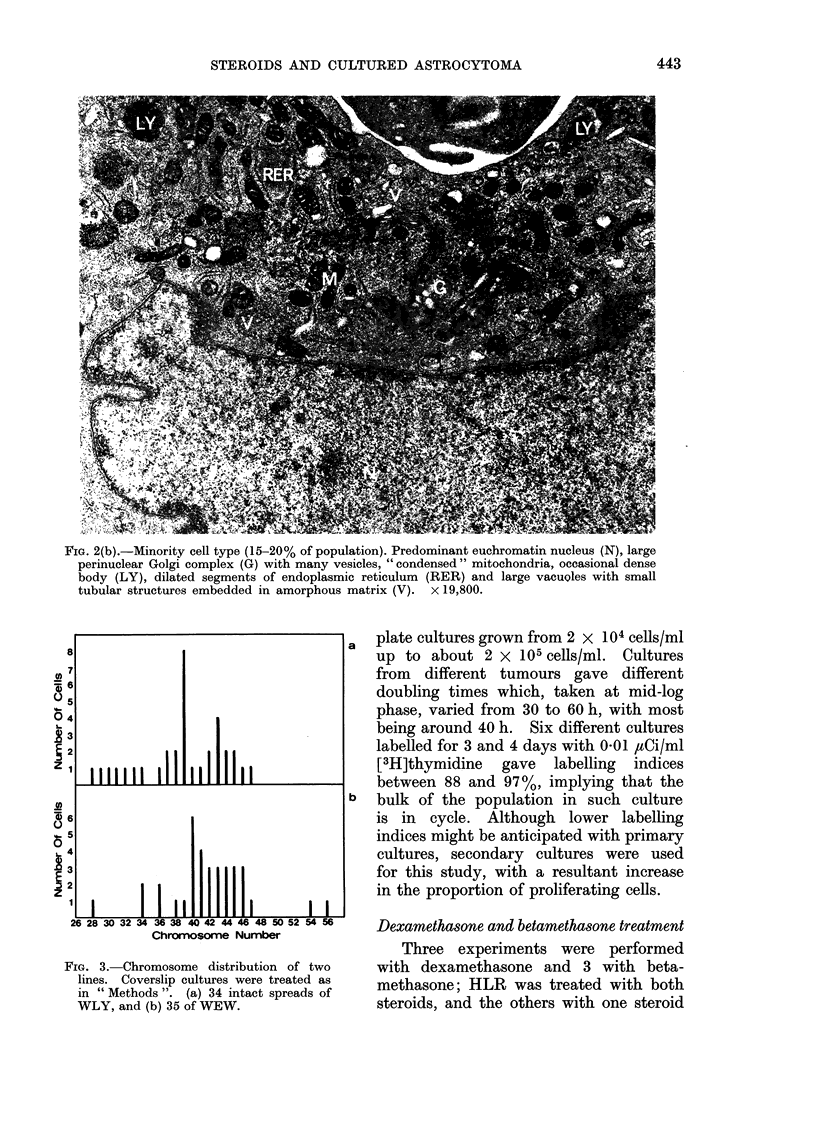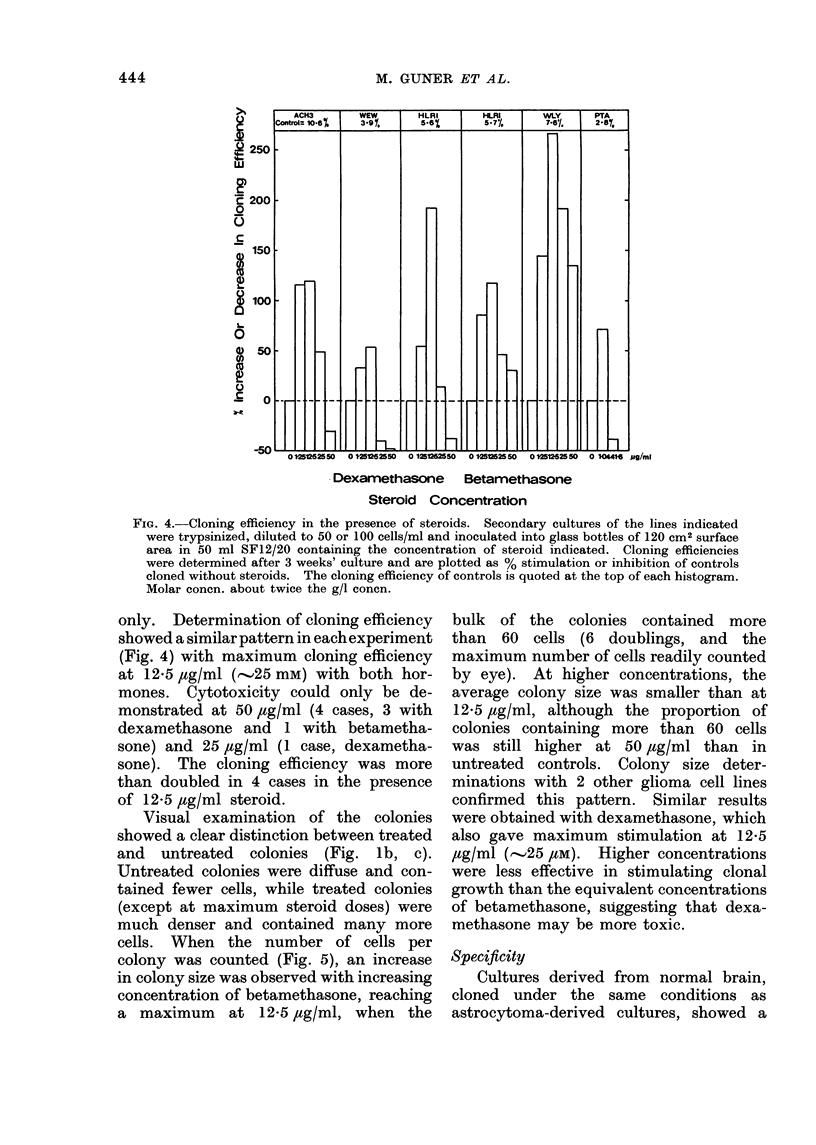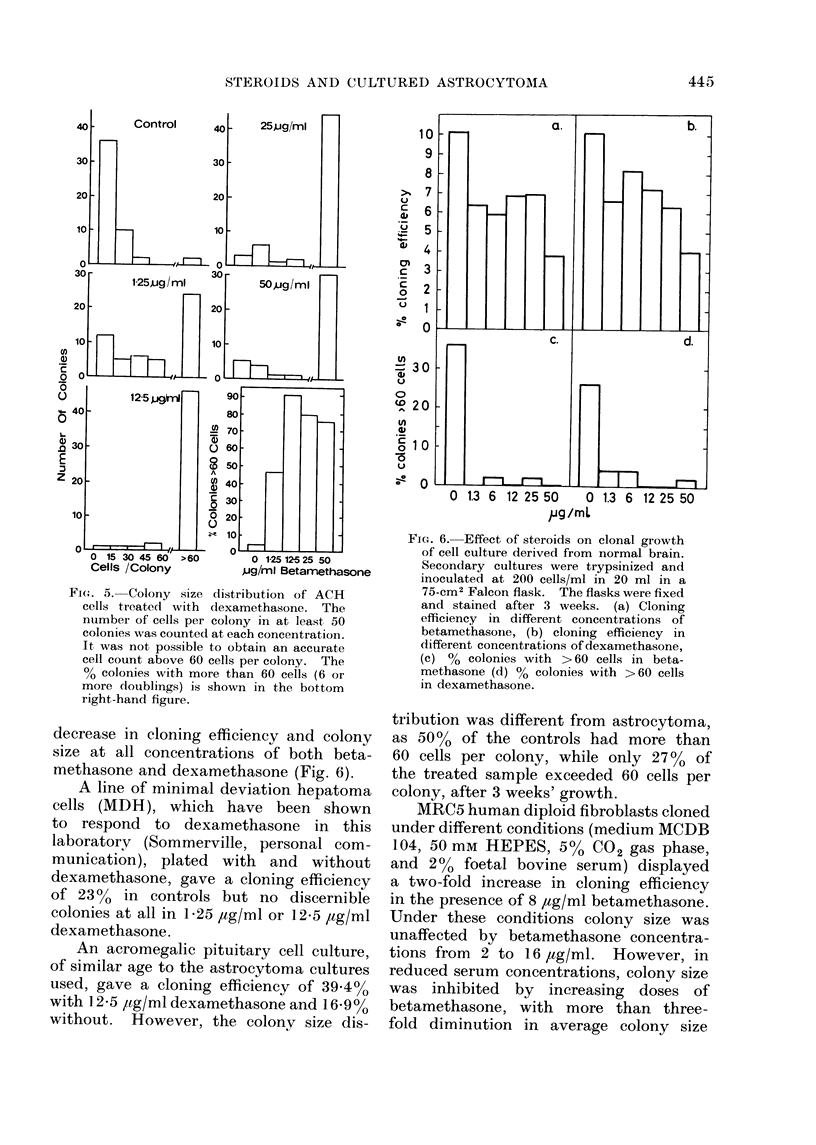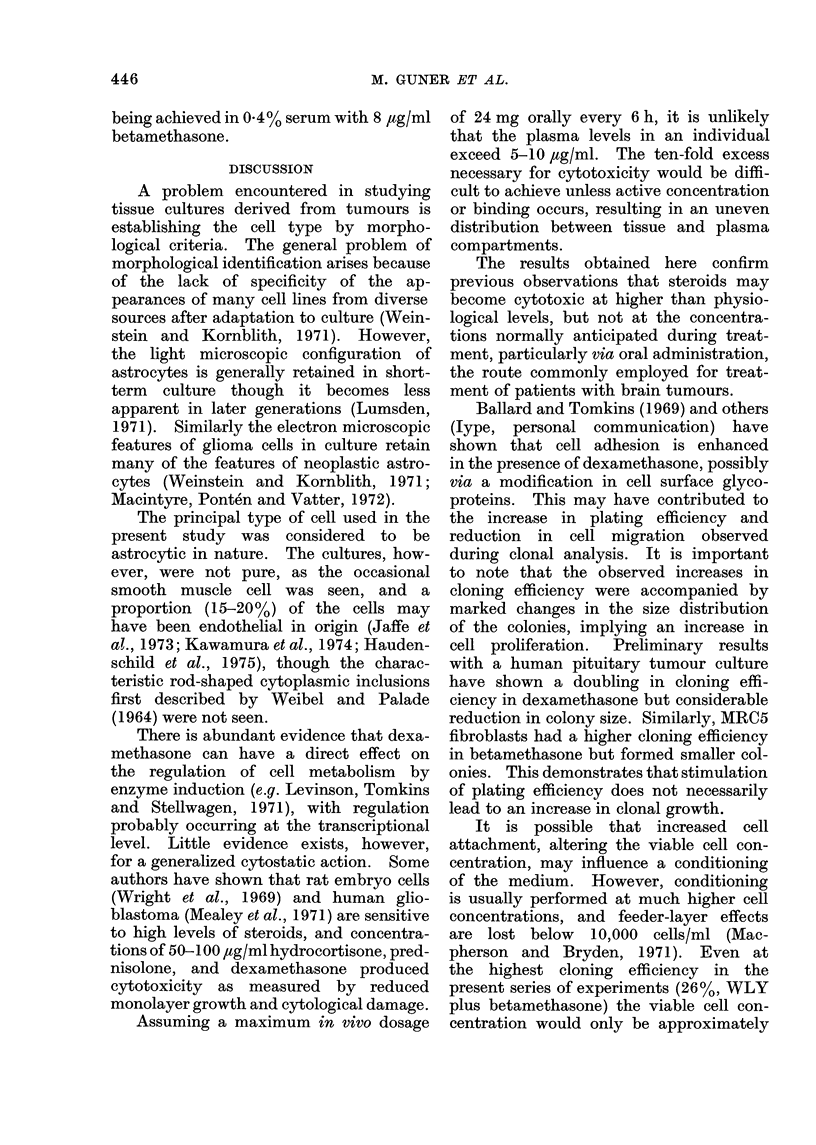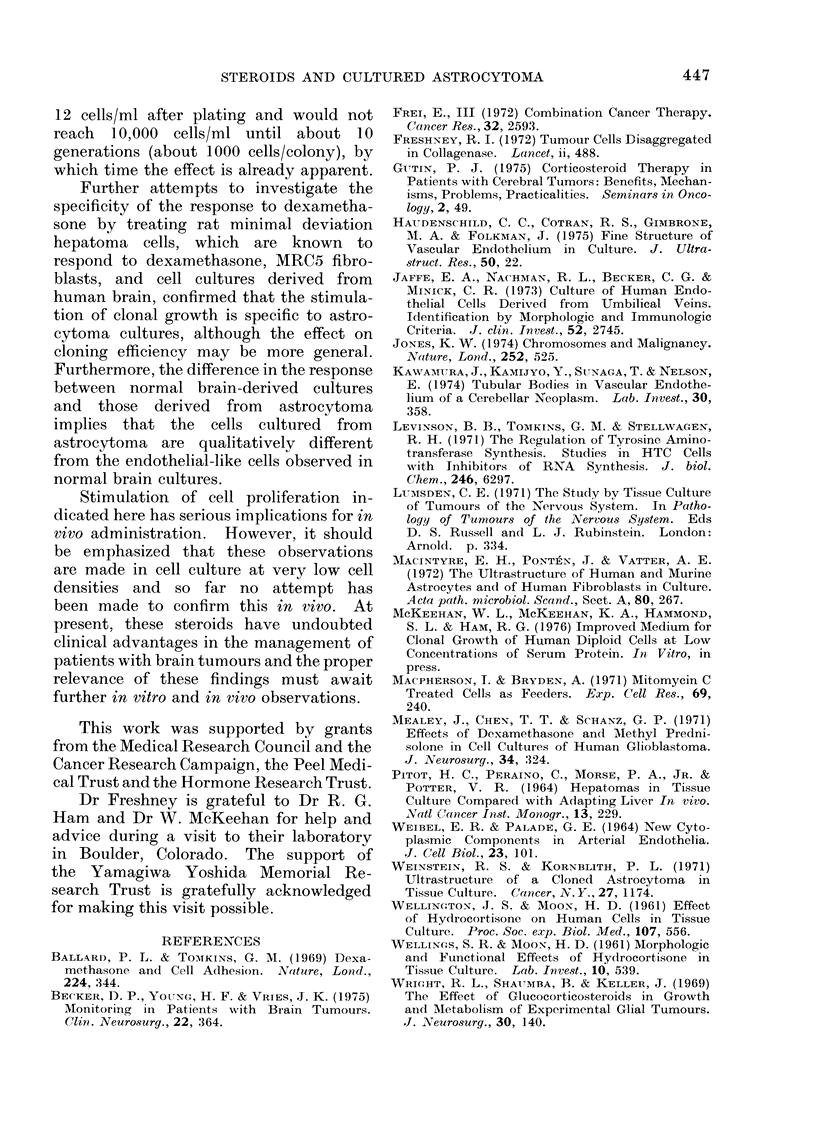# Effects of dexamethasone and betamethasone on in vitro cultures from human astrocytoma.

**DOI:** 10.1038/bjc.1977.66

**Published:** 1977-04

**Authors:** M. Guner, R. I. Freshney, D. Morgan, M. G. Freshney, D. G. Thomas, D. I. Graham

## Abstract

**Images:**


*Br. J. Cancer* (1977) **35**, 439

### EFFECTS OF DEXAMETHASONE AND BETAMETHASONE ON *IN VITRO* CULTURES FROM HUMAN ASTROCYTOMA

M. GUNER*, R. I. FRESHNEY, D. MORGAN, M. G. FRESHNEY, D. G. T. THOMAS AND D. I. GRAHAM

*From the Beatson Institute for Cancer Research, Bearsden, Glasgow, and the Institute of Neurological Sciences, Glasgow*

Received 12 October 1976 Accepted 15 December 1976

**Summary.—Cultures of human astrocytoma have been derived by collagenase digestion and are presumed, from their aneuploid karyotypes, to be predominantly neoplastic. Early passage cultures in proliferative phase have been cloned in the presence of dexamethasone and betamethasone, both commonly used in management of patients with brain tumours. These steroids raise both the cloning efficiency and the proliferative capacity of cells within each clone. Inhibition was detected only in very high steroid concentrations (25–50 μg/ml). Since these concentrations are unlikely to be attained *in vivo* it is concluded that anticipated physiological levels of these steroids enhance cell survival at low densities in culture. The significance of this *in vivo* is discussed.**

ONE of the many clinical applications of glucocorticoid steroids has been in the management of patients with brain tumours. Administration of dexamethasone or betamethasone can rapidly alleviate symptoms due to raised intracranial pressure in these patients, by reduction of cerebral oedema in normal brain surrounding the tumour (Becker, Young and Vries, 1975). Steroid treatment has not only reduced the operative mortality but also appears to improve the quality of life and survival for several months after surgery (Gutin, 1975). Withdrawal of steroids often results in rapid deterioration and death. This has been taken as evidence for a secondary cytostatic action of these drugs, although no kinetic evidence is available *in vitro* or *in vivo* to confirm this.

Well documented evidence exists that steroids are effective, alone and in combination with other drugs, in chemotherapy of leukaemia (Frei, 1972) and Wright, Shaumba and Keller (1969) were able to demonstrate tumour retardation in rats with a transplantable ependymoma treated with methyl prednisolone acetate. A number of reports have shown that levels of 50–100 μg/ml hydrocortisone are cytotoxic to rat embryo (Wellings and Moon, 1961) and Chang liver cells (Wellington and Moon, 1961) but this is so much higher than the anticipated plasma levels that its significance is not clear. Both inhibition and stimulation of proliferation have been described in monolayer cultures of human glioblastoma (Mealey, Chen and Schanz, 1971) but again, significant cytotoxicity was demonstrated mainly above 25 μg/ml dexamethasone phosphate. It remains unclear whether steroids at physiological or chemotherapeutic levels are cytotoxic or cytostatic.

In this report we have attempted to find out whether the two steroids most commonly used with brain tumours, dexamethasone and betamethasone, have any cytostatic effect on clonal growth of human astrocytoma.

* Present address: Gevher Nesibe, Tip Facultesi, Kayseri, Turkey.

### MATERIALS AND METHODS

Cultures were derived from 5 different astrocytomas (Table) by dissociation in collagenase (Freshney, 1972). Aseptically collected biopsy specimens were chopped finely, washed and transferred to culture flasks. Growth medium (Ham's F12 plus Eagle's MEM amino acids (Flow Laboratories), non-essential amino acids (Flow Laboratories) and supplemented with 20% foetal bovine serum (Gibco-Biocult)) was added to give approximately 5–20 mg tissue per ml. Since primary culture and subsequent cloning were performed in sealed bottles with air as gas phase, and buffering was achieved by 20 mM HEPES, the bicarbonate concentration was low (4 mM at the time of preparation). Collagenase (CLS grade, Worthington) was added at 200 u/ml final concentration, and the tissue fragments were incubated for 24–48 h at 36·5°C. The tissue was then dissociated with gentle pipetting, centrifuged to remove collagenase, and resuspended in fresh growth medium at $5 \times 10^4$ to $5 \times 10^5$ cells/ml, depending on the yield. The cells usually attached within 24–48 h and were ready for trypsinization in about 1–2 weeks. Cells for assay were taken after the second trypsinization (1–2 weeks after the first) and were cloned by diluting to 50–100 cells/ml and inoculating 120-cm² glass bottles with 2500–5000 cells per bottle in 50 ml growth medium containing a range of concentrations of dexamethasone or betamethasone. Acromegalic pituitary and normal brain cultures were treated as for astrocytoma. MDH cells (rat minimal deviation hepatoma (Pitot *et al.*, 1964) were grown in the same medium and cloned at 25 cells/ml in 50-cm² glass bottles. MRC5 cells did not clone satisfactorily under these conditions, and were cloned in 5-cm Petri dishes (Lux) in medium MCDB104 (McKeehan *et al.*, 1976) with 2% foetal bovine serum (Flow Laboratories).

TABLE

| Age | Sex | Culture designation | Histological classification |
|---|---|---|---|
| 24 | F | ACH | Anaplastic astrocytoma |
| 45 | M | WEW | Anaplastic astrocytoma |
| 68 | F | HLR | Anaplastic astrocytoma |
| 60 | M | WLY | Anaplastic astrocytoma |
| 25 | M | PTA | Intermediate grade astrocytoma |

Dexamethasone was obtained from Merck, Sharpe & Dohme Ltd as Decadron. Betamethasone as Betnesol was obtained from Glaxo Laboratories Ltd. They were both diluted in Hanks' balanced salt solution and added directly to the culture medium. No addition was made to controls.

Clonal growth cultures were maintained at 36·5°C without medium change for 2–3 weeks. They were then washed in Hanks' BSS, fixed for 10 min in methanol and stained in Giemsa for 10 min. The colonies were counted under a dissecting microscope using a prepared mask with three standard fields of 900 mm² at different parts of the bottle. All the colonies within each field were counted and an extrapolation made to the total surface of the flask. This figure was then used to calculate the cloning efficiency

$$\left(\frac{\text{number of colonies per bottle}}{\text{number of cells inoculated}} \times 100\right).$$

A microscope was used to count colonies, to overcome the difficulty of counting small diffuse colonies found particularly in controls. Only colonies over 16 cells were scored. This figure was chosen arbitrarily to include the product of at least 4 cell generations and avoid problems arising from counting smaller colonies which may later abort.

Colony size determinations were performed by counting the cells per colony in approximately 50 colonies from different parts of the flask. This was done in one experiment with dexamethasone and in three with betamethasone, but all experiments performed with astrocytoma cells have shown increases in colony size which are readily detected by the naked eye.

Chromosome preparations were made by treating coverslips bearing cells in exponential growth with 0·004% colchicine for 4 h. They were then fixed in acetic methanol, dried and stained with Giemsa and counted. At least 20 spreads were counted for each cell strain.

Autoradiographs were performed on coverslip cultures grown in Leighton tubes and labelled for up to 96 h with [$^3$H] thymidine, 0·01 $\mu$Ci/ml (2·0 Ci/mmol). They were washed in BSS, fixed in methanol and dried. After mounting the coverslips on slides, acid-soluble precursors were extracted in 10% ice-cold trichloroacetic acid and the slides rewashed thoroughly in cold distilled water.

Stripping film (Kodak AR-10) was applied and the slides exposed for 3 weeks at 4 °C. They were developed in Kodak D19 for 10 min, washed, fixed in Ilford Hypam for 2 min, washed for 15 min and dried. They were then stained in Giemsa and the percentage labelled cells determined.

*Electromicroscopy.*—After the second trypsinization, about $5 \times 10^6$ cells were fixed with chilled 2% glutaraldehyde in 0·2 M cacodylate buffer for 20 min. They were rinsed in the same buffer prior to post-fixation with cacodylate-buffered 1% osmium tetroxide for 1 h, dehydration in graded ethanol solutions, clearing in propylene oxide, and embedding as pellets in araldite. Thin sections were cut with a glass knife on a LKB ultramicrotome, stained with uranyl acetate and lead citrate, and examined in a Philips EM 201 electron microscope.

### RESULTS

### *Identity of cells*

All cultures were derived from astrocytoma by collagenase digestion. Although cultures have been obtained by us from normal brain by this method, it is unusual to obtain a yield or initial growth rate comparable to that found with tumour material. The cytology of the cells used for clonal growth studies was typical of astrocytoma strains cultured here. The cells were spindle-shaped with very long processes forming a reticular network (Fig. 1a). There was no indication of fibroblastic contamination.

Electron micrographs showed that the majority of the cells in culture were morphologically similar (Fig. 2a and inset). The nucleus was irregular in outline, with clumps of heterochromatin attached to the inner margin of the nuclear membrane, and there was often a prominent nucleolus. The cytoplasm of these cells contained a Golgi complex, moderate quantities of smooth and rough endoplasmic reticulum, a variable number of multivesicular bodies and free ribosomes, 7–10-nm-thick filaments, the occasional dense body and multiple " condensed " mitochondria. Pinocytotic vesicles were not prominent and tubular bodies were not seen.

A minority (15–20%) of the cells contained large numbers of vesicles, a prominent perinuclear Golgi, clusters of

FIG. 1.—Cultures derived from human astrocytoma. (a) Living primary culture 1 week after dissociation in collagenase. Phase contrast. (b) Part of a clone in a low density secondary culture. Giemsa stained. (c) Similar culture treated with 10 μg/ml betamethasone. (a) ×70, (*b*) and (*c*) ×30.

FIG. 2(a).—Electron microscopy of secondary cultures from astrocytoma biopsies. Uranyl acetate and lead citrate. Majority cell type. Cells varied in size and shape. The nucleus (N) often contained a nucleolus and the cytoplasm a perinuclear Goldi complex (G), " condensed " mitochondria (M), segments of endoplasmic reticulum (RER), dispersed ribosomes and multivesicular bodies (MV). ×16,500. Inset Cytoplasm of similar cell showing an abundance of interlacing filaments (F), Mitochondrion = M. ×25,000.

free ribosomes, bundles of fine filaments and " condensed " mitochondria. In some, the cisternae of the endoplasmic reticulum were dilated and contained granular material. Although none of the cells contained rod-shaped cytoplasmic inclusions, large unit membrane-bound vacuoles with a relatively electron-lucent matrix and containing a few tubules were seen in this component (Fig. 2b).

Only the occasional smooth-muscle cell was seen and there appeared to be a complete absence of fibroblasts. The cytoplasm of most cells contained moderately sized clumps of glycogen.

The chromosome numbers of two lines are presented in Fig. 3. Although diploid cells are present, there is evidence of considerable aneuploidy, with chromosome numbers ranging from 28 to 56. Chromosome counts were not available from all the cultures, but 5 others examined here have shown similar hypodiploid distributions to those reported above. Although these results are not conclusive, there is considerable evidence elsewhere that normal human cells remain diploid in culture while neoplastic cells become aneuploid (Jones, 1974). Chromosomal analysis of a culture from normal brain handled in exactly the same manner as the astrocytoma cultures gave a modal number of 46 with a greatly reduced spread.

*Growth potential of secondary cultures*

Population doubling times have been estimated with 10 different secondary cultures from gliomas by counting the number of cells per well in microtitration

FIG. 2(b).—Minority cell type (15–20% of population). Predominant euchromatin nucleus (N), large perinuclear Golgi complex (G) with many vesicles, "condensed" mitochondria, occasional dense body (LY), dilated segments of endoplasmic reticulum (RER) and large vacuoles with small tubular structures embedded in amorphous matrix (V). ×19,800.

FIG. 3.—Chromosome distribution of two lines. Coverslip cultures were treated as in "Methods". (a) 34 intact spreads of WLY, and (b) 35 of WEW.

plate cultures grown from 2 × 10⁴ cells/ml up to about 2 × 10⁵ cells/ml. Cultures from different tumours gave different doubling times which, taken at mid-log phase, varied from 30 to 60 h, with most being around 40 h. Six different cultures labelled for 3 and 4 days with 0·01 $\mu$Ci/ml [$^3$H]thymidine gave labelling indices between 88 and 97%, implying that the bulk of the population in such culture is in cycle. Although lower labelling indices might be anticipated with primary cultures, secondary cultures were used for this study, with a resultant increase in the proportion of proliferating cells.

*Dexamethasone and betamethasone treatment*

Three experiments were performed with dexamethasone and 3 with betamethasone; HLR was treated with both steroids, and the others with one steroid

FIG. 4.—Cloning efficiency in the presence of steroids. Secondary cultures of the lines indicated were trypsinized, diluted to 50 or 100 cells/ml and inoculated into glass bottles of 120 cm² surface area in 50 ml SF12/20 containing the concentration of steroid indicated. Cloning efficiencies were determined after 3 weeks' culture and are plotted as % stimulation or inhibition of controls cloned without steroids. The cloning efficiency of controls is quoted at the top of each histogram. Molar concn. about twice the g/l concn.

only. Determination of cloning efficiency showed a similar pattern in each experiment (Fig. 4) with maximum cloning efficiency at 12·5 μg/ml (~25 mM) with both hormones. Cytotoxicity could only be demonstrated at 50 μg/ml (4 cases, 3 with dexamethasone and 1 with betamethasone) and 25 μg/ml (1 case, dexamethasone). The cloning efficiency was more than doubled in 4 cases in the presence of 12·5 μg/ml steroid.

Visual examination of the colonies showed a clear distinction between treated and untreated colonies (Fig. 1b, c). Untreated colonies were diffuse and contained fewer cells, while treated colonies (except at maximum steroid doses) were much denser and contained many more cells. When the number of cells per colony was counted (Fig. 5), an increase in colony size was observed with increasing concentration of betamethasone, reaching a maximum at 12·5 μg/ml, when the bulk of the colonies contained more than 60 cells (6 doublings, and the maximum number of cells readily counted by eye). At higher concentrations, the average colony size was smaller than at 12·5 μg/ml, although the proportion of colonies containing more than 60 cells was still higher at 50 μg/ml than in untreated controls. Colony size determinations with 2 other glioma cell lines confirmed this pattern. Similar results were obtained with dexamethasone, which also gave maximum stimulation at 12·5 μg/ml (~25 μM). Higher concentrations were less effective in stimulating clonal growth than the equivalent concentrations of betamethasone, suggesting that dexamethasone may be more toxic.

*Specificity*

Cultures derived from normal brain, cloned under the same conditions as astrocytoma-derived cultures, showed a

FIG. 5.—Colony size distribution of ACH cells treated with dexamethasone. The number of cells per colony in at least 50 colonies was counted at each concentration. It was not possible to obtain an accurate cell count above 60 cells per colony. The % colonies with more than 60 cells (6 or more doublings) is shown in the bottom right-hand figure.

FIG. 6.—Effect of steroids on clonal growth of cell culture derived from normal brain. Secondary cultures were trypsinized and inoculated at 200 cells/ml in 20 ml in a 75-cm² Falcon flask. The flasks were fixed and stained after 3 weeks. (a) Cloning efficiency in different concentrations of betamethasone, (b) cloning efficiency in different concentrations of dexamethasone, (c) % colonies with >60 cells in betamethasone (d) % colonies with >60 cells in dexamethasone.

decrease in cloning efficiency and colony size at all concentrations of both betamethasone and dexamethasone (Fig. 6).

A line of minimal deviation hepatoma cells (MDH), which have been shown to respond to dexamethasone in this laboratory (Sommerville, personal communication), plated with and without dexamethasone, gave a cloning efficiency of 23% in controls but no discernible colonies at all in 1·25 µg/ml or 12·5 µg/ml dexamethasone.

An acromegalic pituitary cell culture, of similar age to the astrocytoma cultures used, gave a cloning efficiency of 39·4% with 12·5 µg/ml dexamethasone and 16·9% without. However, the colony size distribution was different from astrocytoma, as 50% of the controls had more than 60 cells per colony, while only 27% of the treated sample exceeded 60 cells per colony, after 3 weeks' growth.

MRC5 human diploid fibroblasts cloned under different conditions (medium MCDB 104, 50 mM HEPES, 5% $CO_2$ gas phase, and 2% foetal bovine serum) displayed a two-fold increase in cloning efficiency in the presence of 8 µg/ml betamethasone. Under these conditions colony size was unaffected by betamethasone concentrations from 2 to 16 µg/ml. However, in reduced serum concentrations, colony size was inhibited by increasing doses of betamethasone, with more than three-fold diminution in average colony size being achieved in 0·4% serum with 8 μg/ml betamethasone.

DISCUSSION

A problem encountered in studying tissue cultures derived from tumours is establishing the cell type by morphological criteria. The general problem of morphological identification arises because of the lack of specificity of the appearances of many cell lines from diverse sources after adaptation to culture (Weinstein and Kornblith, 1971). However, the light microscopic configuration of astrocytes is generally retained in short-term culture though it becomes less apparent in later generations (Lumsden, 1971). Similarly the electron microscopic features of glioma cells in culture retain many of the features of neoplastic astrocytes (Weinstein and Kornblith, 1971; Macintyre, Pontén and Vatter, 1972).

The principal type of cell used in the present study was considered to be astrocytic in nature. The cultures, however, were not pure, as the occasional smooth muscle cell was seen, and a proportion (15–20%) of the cells may have been endothelial in origin (Jaffe *et al.*, 1973; Kawamura *et al.*, 1974; Haudenschild *et al.*, 1975), though the characteristic rod-shaped cytoplasmic inclusions first described by Weibel and Palade (1964) were not seen.

There is abundant evidence that dexamethasone can have a direct effect on the regulation of cell metabolism by enzyme induction (*e.g.* Levinson, Tomkins and Stellwagen, 1971), with regulation probably occurring at the transcriptional level. Little evidence exists, however, for a generalized cytostatic action. Some authors have shown that rat embryo cells (Wright *et al.*, 1969) and human glioblastoma (Mealey *et al.*, 1971) are sensitive to high levels of steroids, and concentrations of 50–100 μg/ml hydrocortisone, prednisolone, and dexamethasone produced cytotoxicity as measured by reduced monolayer growth and cytological damage.

Assuming a maximum *in vivo* dosage of 24 mg orally every 6 h, it is unlikely that the plasma levels in an individual exceed 5–10 μg/ml. The ten-fold excess necessary for cytotoxicity would be difficult to achieve unless active concentration or binding occurs, resulting in an uneven distribution between tissue and plasma compartments.

The results obtained here confirm previous observations that steroids may become cytotoxic at higher than physiological levels, but not at the concentrations normally anticipated during treatment, particularly *via* oral administration, the route commonly employed for treatment of patients with brain tumours.

Ballard and Tomkins (1969) and others (Iype, personal communication) have shown that cell adhesion is enhanced in the presence of dexamethasone, possibly *via* a modification in cell surface glycoproteins. This may have contributed to the increase in plating efficiency and reduction in cell migration observed during clonal analysis. It is important to note that the observed increases in cloning efficiency were accompanied by marked changes in the size distribution of the colonies, implying an increase in cell proliferation. Preliminary results with a human pituitary tumour culture have shown a doubling in cloning efficiency in dexamethasone but considerable reduction in colony size. Similarly, MRC5 fibroblasts had a higher cloning efficiency in betamethasone but formed smaller colonies. This demonstrates that stimulation of plating efficiency does not necessarily lead to an increase in clonal growth.

It is possible that increased cell attachment, altering the viable cell concentration, may influence a conditioning of the medium. However, conditioning is usually performed at much higher cell concentrations, and feeder-layer effects are lost below 10,000 cells/ml (Macpherson and Bryden, 1971). Even at the highest cloning efficiency in the present series of experiments (26%, WLY plus betamethasone) the viable cell concentration would only be approximately

12 cells/ml after plating and would not reach 10,000 cells/ml until about 10 generations (about 1000 cells/colony), by which time the effect is already apparent.

Further attempts to investigate the specificity of the response to dexamethasone by treating rat minimal deviation hepatoma cells, which are known to respond to dexamethasone, MRC5 fibroblasts, and cell cultures derived from human brain, confirmed that the stimulation of clonal growth is specific to astrocytoma cultures, although the effect on cloning efficiency may be more general. Furthermore, the difference in the response between normal brain-derived cultures and those derived from astrocytoma implies that the cells cultured from astrocytoma are qualitatively different from the endothelial-like cells observed in normal brain cultures.

Stimulation of cell proliferation indicated here has serious implications for *in vivo* administration. However, it should be emphasized that these observations are made in cell culture at very low cell densities and so far no attempt has been made to confirm this *in vivo*. At present, these steroids have undoubted clinical advantages in the management of patients with brain tumours and the proper relevance of these findings must await further *in vitro* and *in vivo* observations.

This work was supported by grants from the Medical Research Council and the Cancer Research Campaign, the Peel Medical Trust and the Hormone Research Trust.

Dr Freshney is grateful to Dr R. G. Ham and Dr W. McKeehan for help and advice during a visit to their laboratory in Boulder, Colorado. The support of the Yamagiwa Yoshida Memorial Research Trust is gratefully acknowledged for making this visit possible.

### REFERENCES

Ballard, P. L. & Tomkins, G. M. (1969) Dexamethasone and Cell Adhesion. *Nature, Lond.*, **224**, 344.

Becker, D. P., Young, H. F. & Vries, J. K. (1975) Monitoring in Patients with Brain Tumours. *Clin. Neurosurg.*, **22**, 364.

Frei, E., III (1972) Combination Cancer Therapy. *Cancer Res.*, **32**, 2593.

Freshney, R. I. (1972) Tumour Cells Disaggregated in Collagenase. *Lancet*, ii, 488.

Gutin, P. J. (1975) Corticosteroid Therapy in Patients with Cerebral Tumors: Benefits, Mechanisms, Problems, Practicalities. *Seminars in Oncology*, **2**, 49.

Haudenschild, C. C., Cotran, R. S., Gimbrone, M. A. & Folkman, J. (1975) Fine Structure of Vascular Endothelium in Culture. *J. Ultrastruct. Res.*, **50**, 22.

Jaffe, E. A., Nachman, R. L., Becker, C. G. & Minick, C. R. (1973) Culture of Human Endothelial Cells Derived from Umbilical Veins. Identification by Morphologic and Immunologic Criteria. *J. clin. Invest.*, **52**, 2745.

Jones, K. W. (1974) Chromosomes and Malignancy. *Nature, Lond.*, **252**, 525.

Kawamura, J., Kamijyo, Y., Sunaga, T. & Nelson, E. (1974) Tubular Bodies in Vascular Endothelium of a Cerebellar Neoplasm. *Lab. Invest.*, **30**, 358.

Levinson, B. B., Tomkins, G. M. & Stellwagen, R. H. (1971) The Regulation of Tyrosine Aminotransferase Synthesis. Studies in HTC Cells with Inhibitors of RNA Synthesis. *J. biol. Chem.*, **246**, 6297.

Lumsden, C. E. (1971) The Study by Tissue Culture of Tumours of the Nervous System. In *Pathology of Tumours of the Nervous System.* Eds D. S. Russell and L. J. Rubinstein. London: Arnold. p. 334.

Macintyre, E. H., Pontén, J. & Vatter, A. E. (1972) The Ultrastructure of Human and Murine Astrocytes and of Human Fibroblasts in Culture. *Acta path. microbiol. Scand.*, Sect. A, **80**, 267.

McKeehan, W. L., McKeehan, K. A., Hammond, S. L. & Ham, R. G. (1976) Improved Medium for Clonal Growth of Human Diploid Cells at Low Concentrations of Serum Protein. *In Vitro*, in press.

Macpherson, I. & Bryden, A. (1971) Mitomycin C Treated Cells as Feeders. *Exp. Cell Res.*, **69**, 240.

Mealey, J., Chen, T. T. & Schanz, G. P. (1971) Effects of Dexamethasone and Methyl Prednisolone in Cell Cultures of Human Glioblastoma. *J. Neurosurg.*, **34**, 324.

Pitot, H. C., Peraino, C., Morse, P. A., Jr. & Potter, V. R. (1964) Hepatomas in Tissue Culture Compared with Adapting Liver *In vivo*. *Natl Cancer Inst. Monogr.*, **13**, 229.

Weibel, E. R. & Palade, G. E. (1964) New Cytoplasmic Components in Arterial Endothelia. *J. Cell Biol.*, **23**, 101.

Weinstein, R. S. & Kornblith, P. L. (1971) Ultrastructure of a Cloned Astrocytoma in Tissue Culture. *Cancer, N.Y.*, **27**, 1174.

Wellington, J. S. & Moon, H. D. (1961) Effect of Hydrocortisone on Human Cells in Tissue Culture. *Proc. Soc. exp. Biol. Med.*, **107**, 556.

Wellings, S. R. & Moon, H. D. (1961) Morphologic and Functional Effects of Hydrocortisone in Tissue Culture. *Lab. Invest.*, **10**, 539.

Wright, R. L., Shaumba, B. & Keller, J. (1969) The Effect of Glucocorticosteroids in Growth and Metabolism of Experimental Glial Tumours. *J. Neurosurg.*, **30**, 140.